# American Dental Association

**Published:** 1892-12

**Authors:** 


### AMERICAN DENTAL ASSOCIATION.

*Second Day.—Morning Session.*

(Continued from page 836.)

THE meeting was called to order by the President, Dr. Walker. The minutes of the previous session were read and approved.

Dr. W. C. Barrett, of Buffalo, then read a paper, entitled "A Brief Study of the Molar Teeth of the Proboscidiæ," of which an abstract follows:

"The student of comparative dental anatomy nowhere, in all the broad realms of nature, finds better opportunities for study than in the teeth of the Pachydermata, or thick-skinned animals, a subdivision of the great order of the Ungulata, and in which the Proboscidians form a suborder. In tracing the modifications of dentition through the different species, it must be remembered that a number are lost, and the regular gradations do not appear.

"But in the pachyderms, as they now exist, or have existed within comparatively modern times, modifications may be traced which are a sure indication of the gradual development of certain species through the lapse of cycles of time. We find families which in their dental development seem to the ordinary observer as widely separated as the East is from the West, but which a close examination proves to be near congeners.

"What analogy, for instance, is there between the tapir, the last remnant of a great Eocene family, with its forty-two teeth in full use at one time, and the elephant, the final existing species of the almost extinct Proboscidians, having only six? And yet the gradations are plainly indicated. The molars of Tapirus have distinctly cone-shaped divisions of the crown. In the Dinotherium these are also found, but the separate denticles have united, and the cone-shaped prominences have become sections, each having its separate root. There are modifications of this change found in certain intermediate forms, which indicate the process of union of the separate teeth, showing that both dentitions had the same common source.

"In the fossil mastodon, named from the shape of its teeth (*μαστὸς*, a nipple, and *οδούς*, a tooth), we find the same general form of dentition, but materially modified as to structure. Finally, in Elephas there is a yet further modification, the cone-shaped prominences being lost, and the separate denticles taking the form of dentinal plates. The structure, too, is changed, but there still remains the body of dentine surrounded by enamel, the whole being united by cementum, the modifications being those which would be induced by the gradual change of the denticles into dentinal plates.

"The six incisors of the tapir we find reduced to four in the mastodon, and of these the inferior ones are rudimentary, while the superior permanent pair, finding no lower ones with which they might occlude, grow to an enormous size and are developed into tusks, precisely as the incisor teeth of rodents grow to a great length when the opposing one is lost by accident.

"In the Dinotherium it is the superior incisors which have become rudimentary, and the lower ones are in the same manner developed into great tusks, preserving their natural outward curvature. In the elephant the incisors follow the law of its congener, the mastodon, keeping the natural curve that is imparted to them by the shape of their sockets, and develop into those enormous tusks that distinguish the species. The study of the incisor teeth of the elephant is a fascinating subject, but the limits of time demand that I should confine myself mainly to a consideration of the molars. Dr. Miller, in the *Dental Cosmos*, has given a very valuable series of papers upon the development and certain pathological disturbances in the tusks of elephants, and to that let me refer the student who desires something further in this direction.

"The dentition of *Mastodon giganteus*, of North America, is a distinct modification between that of the Dinotherium and the elephant. A part of the molars are succeeded by vertical successors, as is usual in the diphyodonts, so that there are, strictly speaking, true premolars. The denticles which make up the compound tooth follow the general law in mammalian tooth-structure, and are formed of a body of dentine covered by enamel, with a root-envelope of cementum.

"In the molar of the true elephant we find a remarkable modification, not only in structure, but in development. The succession, instead of being vertical, is horizontal. The formula is as follows: $\frac{2\text{–}2}{0\text{–}0}\ \text{m.}\ \frac{6\text{–}6}{6\text{–}6} = 28.$ Of the incisors, two are deciduous, while the permanent ones form the great tusks. The molars of the elephant are of great proportionate size, especially those which make their appearance later in life. There is never but one on each side of the jaw in full use at any time, and all through life there is going on the constant process of the shedding of this and the formation and advance of its successor from behind. The food of the elephant is of an exceedingly coarse character, and although the structure of the molars is such as to offer the greatest resistance to attrition, yet no other form of dentition would be sufficient for the long life of the animal.

"The germs of the six molars on each side of each jaw exist within the body of the maxillæ, and are developed in turn as the predecessor is worn out and shed. The structure is very complex, and is shown in the chart which I exhibit. The teeth are made up of transverse, perpendicular plates, which are developed separately within a crypt in the jaw. The size of these plates depends somewhat upon the age of the animal. I exhibit to you the bisected jaw of an adult elephant, and as I open it you are able to see the crypt or chamber which contains these developing plates. Each of them consists of a body of dentine, surrounded by an enamel covering. The development is from these elevated points, as shown in this figure, which give to the forming plates a serrated appearance, not unlike that sometimes seen in the newly-erupted incisors of children. These plates are open at the base, the cavity being occupied by the pulp-tissue from which the dentine is developed.

"I have not had the opportunity to examine any fresh specimens microscopically, so that it is impossible for me to say in just what manner the enamel is formed; but as that tissue covers the whole plate, and as during the process of formation these are separated by a considerable space, it is probable that there is a separate enamel organ for each one, and that this continues its function to the time when the plates coalesce into the complete tooth. In the rooms of the Odontological Society of New York there is the complete skull of an elephant, the jaws bisected as are some of these now before you, but in which the separate plates are in a more advanced condition than are these, and which therefore more fully illustrate the formation, in that the plates are perhaps three times as large as are the ones I now show you.

"The growth of these plates continues until the enamel membrane is exhausted, when there is, from the several divisions of the pulp, a further deposition of dentine, which forms a strong base for the whole, uniting the plates into a solid body. As the tooth after eruption is worn away, roots are developed, which serve to retain it in position. In its earlier stages it is held by the great body being within the socket.

"Coincident with the formation of the great body of dentine at the base, the enveloping membrane begins the deposition of cementum between and about the separate plates, thus uniting their upper portions into a solid body as the dentine unites the base, and in this manner is formed that complex structure illustrated in the diagrams exhibited, as well as by the numerous prepared sections upon the table before me. There is, then, here in the centre of each plate, a body of dentine surrounded by a complete ring of enamel, and uniting the separate plates a stratum of cementum, thus presenting a yet more perfect arrangement of alternate dentine, enamel, and cementum than is found in the molars of the more common Graminivora. The several tissues offering a different degree of resistance, the teeth are worn into the alternate ridges and depressions that offer the most perfect surface for the proper comminution of coarse food.

"As the deposition of the enamel about the body of the dentine in each separate plate proceeds, it assumes a wavy or undulating appearance. In the Indian elephant, and in the extinct Mammoth, the sides of the enamel-walls are parallel, thus causing the enamel to present the appearance of a complete and regular ring. In the African elephant the body of the dentine is much thicker in the centre, and the enamel covering is expanded until there may be a complete separation in the middle of the ring, giving to the dentine a lozenge shape, and to the enamel of each lateral half of the tooth a zigzag appearance. This is very apparent in some of the teeth now before you, and by this peculiarity of structure it is comparatively easy to determine to which species each belonged. In the Mammoth the plates are thinner and more numerous, as may be observed in this specimen.

"In the procession of the teeth, a part of the posterior one comes into use before its predecessor is thrown off, for the whole process is a gradual one. While all the posterior plates are covered by the gum and bony tissues, the anterior ones are in use. Here is a tooth in which it may be seen that the first three or four plates are considerably worn, while the posterior ones bear no such marks. In the lower jaw the forming teeth lie within the ramus, while in the upper they are within the retreating portion. In the course of their descent, therefore, following the general contour of the jaw, they describe the arc of a circle, instead of advancing in a straight line. This peculiar conformation, therefore, presents the anterior angle of the tooth first. As it gradually descends from above and reaches the plane of the body of the jaw, the tooth assumes a horizontal position, and the successive plates are brought into full use. Before its eruption it stands almost at a right angle to its predecessor.

"This peculiar method of progression forms one of the most striking characteristics of the dentition. It brings the plates successively into use, and by the time the last one is in full action the anterior ones are considerably worn away. The tooth will then have a wedge shape, and its anterior border would have but a comparatively slight hold in the jaw, were it not that this is compensated by the development of the anterior roots. In the course of the attrition the foremost plates become worn down to the dentinal base and are smooth, while the posterior ones yet have their enamel intact. This serves a useful purpose in mastication, for, as Owen remarks, the coarse limbs and twigs of trees are broken up and crushed on the anterior smooth plates, and thoroughly comminuted between the deeply-ridged posterior ones.

"When the tooth becomes worn down so far that the basal mass of dentine is seriously interfered with, the pulp seems to lose its functional power, and becomes exhausted. A reversal of the process now sets in, and there is resorption of the roots, until the advance of its successor crowds it forward and finally out of the jaw, the other taking its place. This process is repeated in the several teeth until the last one appears, and this is sufficient for the needs of the animal until it dies from old age.

"Each successive tooth presents an increased number of plates, and therefore lasts for a longer time than its predecessor. The sixth molar seems to develop more slowly than any of the others, and to have an unusual number of plates, which come into use with increasing tardiness. It would seem that in an animal that lives to an extraordinary age the plates are continually added, that the tooth may serve for mastication until death. Here is such a one. The animal to which this belonged was probably considerably past the half-century mark, and yet it will be seen that there are many years of service left in the organ. While the first plates show the attrition produced by actual use, the last ones are yet in the process of formation, being quite immature. Of the twenty-odd plates, only ten have as yet been brought into action, and some of these are not worn down to the solid enamel. There was here a dental provision for many years of life.

"I have here the skull of a young Indian elephant, in which the points of the deciduous tusks are but just discernible. The exact age of the animal I have not been able to determine, any further than by the appearance of the skull. But it will be observed that the tissue around the extremities of the tusks is in a cartilaginous condition, and that they had not yet protruded through the gum, which should occur between the fifth and the sixth month. Yet the first molar is completely erupted, and shows some attrition, so that it is probable that the young animal at death was three or four months old. The bones of the head, while firmly united, are yet very frail, and the zygomatic processes but partially ossified. The first molars, which cut the gum in the second week, with portions of the second, are plainly visible, and illustrate the formation of the teeth very perfectly.

"In the upper jaw the first molar has six plates, while in the lower there are seven. The anterior plates of the second molar were not yet erupted, but they are completely united by cementum, while the posterior ones are only held by the dentine at their base. The crypt for the third molar may be distinctly observed, although the plates are not sufficiently developed to be plainly seen. But the progression through the arc of a circle is beautifully illustrated, and the manner in which they are brought into use.

"Owen says that the first molar, which is in complete use at three months, and is shed at about two years of age, usually has four plates, but in this specimen there are more. The second molar, according to the same authority, has eight or nine plates, and that is about the number in this. He gives the length of the first molar at less than an inch, while in this specimen they are fully two inches. The second molar, he says, is about two and a half inches in its antero-posterior diameter. In this skull, although not fully developed, it is more than three inches. It may thus be seen that there is no determinate number of plates in different individuals, but that it varies with the development.

"The third molar has from eleven to thirteen plates, and is shed during the ninth year. The plates of the fourth molar number fifteen or sixteen, and it is shed from the twentieth to the twenty-fifth year. The fifth molar has from seventeen to twenty plates, and is probably not shed until the sixtieth year. Thus the first molar lasts but about two years. The second remains about four years, the third about the same time, the fourth eleven or twelve years, the fifth thirty years, while the duration of the sixth has not been definitely determined. The plates of the sixth molar are from twenty-two to twenty-seven, and it is from twelve to fifteen inches in length. . . .

"The advance in the grinding teeth is accompanied by a like advance in the alveolus, which is developed with them. The tooth, as it emerges from the body of the jaw, is enveloped in the cancellous bone which forms the socket, and as it moves forward there is a corresponding progression of the alveolus which retains it, and which is finally resorbed with the roots of the teeth as they reach the most anterior point, thus allowing their final removal. At the same time there is a new formation about the succeeding molar, which passes through the same stages. It would be difficult to conceive how the progression of the teeth could be accomplished by any other means.

"Although there are no deciduous molars, the elephant is provided with milk incisors, or tusks, which grow to the length of but a few inches, and are shed between the first and second year. They are almost immediately succeeded by the permanent ones, which, arising from persistent pulps, like the incisors of the Rodentia, like them continue to grow through life.

"Abnormities in the development of the teeth of these great mammals are not unknown. Especially are the incisors or tusks liable to irregularities. Not infrequently is their direction changed, through some injury to the alveolus. I have more than once seen captive elephants, the points of whose tusks crossed each other. Occasionally one tusk is considerably more elevated than the other. To a certain extent they are movable in their sockets, and a continuous pressure upon them will change their direction and relative position. These irregularities may be corrected, when the animal is not too old, by any force continuously applied, precisely as the human teeth are made to assume new positions by artificial means.

"In the rooms of the New York State Agricultural Society, at Albany, is the skeleton of the celebrated Cohoes mastodon, which was dug up some years since. The inferior jaw shows the loss of one of the molars, probably through some accident, and the alveolus has completely closed over the place. Probably the animal was not a very old one, but there are no signs of another molar beneath it. If it were possible to make a section of the jaw, perhaps something of interest might be discovered. I noticed this peculiarity some years ago, but have never seen it mentioned by any author. I have not had the opportunity to make a very thorough examination of the case, something which 1 propose to do in the near future.

"The most singular malformation that I have ever observed in any animal appears in the upper jaw of the celebrated elephant 'Jumbo,' which was killed in a railroad accident a few years since. This was an African elephant of prodigious size, although it had scarcely reached the age of full maturity. Captive African elephants are rare, the most of those in menageries being of the Asiatic species. The skeleton of 'Jumbo' was mounted at the Natural Science Establishment of Professor Henry A. Ward, of Rochester, N. Y., for Barnum & Bailey, the showmen proprietors of 'Jumbo' when alive, and is deposited in the rooms of the Society of Natural Sciences, in Central Park, New York City. I visited the skeleton a few months since, and called the attention of the superintendent to the many points of interest it presented, suggesting the advisability of an examination by making sections of the jaw. I was informed that as it was not the property of the Society this could not be done, but they would endeavor to obtain the necessary permission of Mr. Bailey to do it.

"I exhibit here casts of the upper jaw, obtained when the skeleton was mounted in Professor Ward's establishment. It will be seen that the aberration was remarkable. There are two molars in each jaw fully erupted. At his death 'Jumbo' was about twenty-five years of age, and this would make of these the fourth in succession, provided the usual rate of progression had been followed. Yet Owen and Corse say that the fourth molar has about fifteen plates, while these have but ten. The same authorities give the length of the tooth at about eight inches, and that is the size of these. So we may probably accept them as the fourth molars.

"None of the visible teeth occupy their normal positions, nor are any of them of the usual form. The right molar is nearest the normal, yet this is curved considerably, the convex surface being outward. Instead of lying longitudinally in the jaw, it stands at an angle of about thirty degrees. The plates, however, are normally developed.

"Lying inside the posterior half of the tooth, and at nearly the same angle with the jaw, are the first five or six plates of the succeeding molar. In the plaster cast it is difficult to determine just what was the situation, but the plates were markedly distorted, some of them lying almost longitudinally, instead of transversely. Those which were not yet fully developed were on a still different plane, the posterior half of the tooth standing at an angle to the rest. Only a section of the jaw would reveal the exact manner of their formation.

"The left fourth molar presents a still greater anomaly, the convex side being inward. At about the junction of the fifth and sixth plates the tooth makes a sharp bend, the posterior plates standing at an angle of nearly ninety degrees to the rest. The developed portion of the left superior fifth molar presses against the deflected posterior plates of its predecessor, in such a manner as to tend to turn it upon its axis. The plates of the fifth molar are irregularly developed, the first and second being nearly an inch apart, and the others separated by a very wide layer of cementum.

"The general aspect of this case is that of the irregular development of the fifth molar without the removal of the fourth, and the malposition of all of them. The fourth molars are worn, the anterior plates being almost reduced to the dentinal base, but there has not apparently been the usual absorption that should take place at this time. The alveolus, especially upon the right side, has been extraordinarily developed, as might naturally have been expected. Those who closely observed the animal in life could not fail to mark the asymmetry in the jaws. I myself noticed it long before I had an opportunity to study it.

"What was the cause of this irregularity? The elephant, although not born in captivity, was captured when very young. He was of extraordinary size, and his whole bony system was unusually massive. He had always been of a peaceful disposition, and had not, until his death, met with any serious accident.

"A duplicate cast of the jaw was sent to Professor W. H. Flower, director of the Natural History Department of the British Museum, author of "The Osteology of the Mammalia," etc., and perhaps as competent authority as now lives upon the subject. He gave his opinion in a letter, in the course of which he says,—

"'The irregularity has arisen evidently from his food not having sufficient gritty particles in it to cause the required and normal wear of the front ones, so that these were not gotten rid of, as they ought to have been, before the others came up to take their places. Elephants in menageries constantly suffer in this way. They ought to have a little sand mixed with their food.'

"Notwithstanding the eminence of the authority, I am inclined to take issue with Professor Flower upon the etiology of this case. I would not assert that the lack of attrition was not a factor in the result, but I cannot believe that alone it is sufficient to account for the aberration, for the following reasons:

"*First.* It is not absolutely certain that the procession of the teeth in Elephas is entirely due to their wear. Certainly that has something to do with it; but if the animal was entirely fed on food that produced no abrasion of their surfaces, it seems to me there can be no doubt that they would be shed. Probably the time might be deferred, but the posterior molars would certainly develop, and to do this the anterior ones must be removed. We know that in the human subject the deciduous teeth are removed with the development of their successors, and that when there are no permanent germs the first ones are usually retained. This would indicate that the absorption of the deciduous roots is due to the development of their successors.

"*Second.* With the wearing away of the crowns there is a development of the roots of the worn teeth in elephants, so that their removal does not depend upon mere attrition,—that, indeed, inducing exactly the contrary tendency. They are absorbed, as are the deciduous teeth in other mammals. It is a part of the mysterious process of nature, influenced by the pressure of the succeeding tooth. That in elephants the eruption and advance is horizontal instead of vertical does not necessarily change the conditions. Lateral pressure is as effective in inducing resorption as is vertical.

"*Third.* I cannot conceive that the lack of wear of the teeth should affect the structure of their successors. Their form might possibly be changed, as the result of undue pressure upon the formative plates; but this would naturally result in their being crowded together, whereas in the teeth of 'Jumbo' the plates are separated by an undue amount of cementum.

"*Fourth.* The relative proportion of the different tissues is completely changed, thus showing that the development within the crypt of the jaw in the early stages was interfered with. Even though the preceding tooth was not advanced it would not induce this aberration, because the anterior plates are formed before the normal time for the advance of its predecessors has arrived.

"*Fifth.* The fourth molars, in the case of 'Jumbo,' seem to have been considerably worn. The fifth molars are occupying a place anterior to that which belongs to them. They have, it seems to me, unduly advanced. The general appearance is that of teeth that have irregularly developed. It is quite impossible to determine the structure of the posterior plates without making a section of the jaw, as they are yet enveloped in the bone.

"*Sixth.* It is difficult to conceive of food that would cause greater attrition than that which is usually supplied to elephants in captivity. In their native condition they subsist largely upon succulent grasses and shrubs, which in their green state are easily masticated. In captivity elephants are mainly fed upon dry hay, than which few foods could wear the teeth faster, and desiccated grains, the siliceous covering of which must cause great wasting of the tissues of the teeth. It would appear, then, that the amount of attrition in the teeth of a captive elephant should be much greater than in those of one which lives in a state of nature.

"In view of these facts, it does not seem to me reasonable to suppose that this abnormity could be caused by lack of gravel in the food of 'Jumbo.' I should rather attribute it to some injury which he had sustained, and which interfered with the development of the tooth-plates. It might be sufficient to accomplish that without being externally visible. The reversed convexity of the teeth, the change in their morphology, the abnormal development of the alveolus, the malarrangement of the tissues, the distortion in the tooth-plate, all seem to point to this as the predisposing cause. It is possible that the jaw itself is but partially developed. It seems short for an animal of such extraordinary size.

"Nothing but an examination of the forming plates, and of the germs of the remaining teeth, will satisfactorily solve this very interesting problem, and it is very much to be hoped that some competent New York comparative dental anatomist will receive the necessary permission, and make a careful study of this unique case."

(To be continued.)

